# Giant Modulation of Interlayer Coupling in Twisted Bilayer ReS_2_

**DOI:** 10.1002/advs.202500411

**Published:** 2025-04-25

**Authors:** Krishna P. Dhakal, Trang Thu Tran, Taegeon Lee, Wooseon Choi, Sean F. Peterson, Juan M. Marmolejo‐Tejada, Jaeuk Bahng, Daekwon Lee, Vu Khac Dat, Ji‐Hee Kim, Seong Chu Lim, Martín A. Mosquera, Young‐Min Kim, Heesuk Rho, Jeongyong Kim

**Affiliations:** ^1^ Department of Energy Science Sungkyunkwan University Suwon 16419 Republic of Korea; ^2^ Department of Physics, Research Institute for Materials and Energy Sciences Jeonbuk National University Jeonju 54896 Republic of Korea; ^3^ Department of Physics Montana State University Bozeman MT 59717 United States; ^4^ Department of Chemistry and Biochemistry Montana State University Bozeman MT 59717 United States; ^5^ Efficient Power Conversion Corp. El Segundo CA 90245 United States; ^6^ Department of Smart Fabrication Technology Sungkyunkwan University Suwon 16419 Republic of Korea; ^7^ Department of Physics Pusan National University Busan 46241 Republic of Korea; ^8^ Center for 2D Quantum Heterostructures Institute for Basic Science (IBS) Suwon 16419 Republic of Korea

**Keywords:** excitons, interlayer coupling, ReS_2,_ twistronics

## Abstract

Stacking monolayers of two‐dimensional (2D) transition metal dichalcogenides with different twist angles can provide a way to tune their quantum optical and electronic characteristics. This study demonstrates that the bandgap energy and interlayer coupling strength of twisted bilayer (tBL) ReS_2_ can be continuously modulated by the twist angle. By controlling the twist angle between 0° and 10°, the exciton energy of tBL ReS_2_ is tuned over a range of 40 meV, which is comparable to the difference between the exciton energies of intrinsic monolayer and bilayer ReS_2_. Such a wide modulation range for the interlayer coupling strength of tBL ReS_2_, which significantly affects the band structure, is also shown by the systematic shift in the low‐and high‐frequency Raman modes and results of a strain study using scanning transmission electron microscopy imaging. Density functional theory calculations on moiré superlattice tBL ReS_2_ structures confirm a consistent increase in the bandgap with the twist angle. The strong modulation of interlayer coupling by the twist angle in tBL ReS_2_ is attributed to the low symmetry of the 1T*'* structure and in‐plane anisotropy of the ReS_2_ lattice. These findings demonstrate the enhanced tunability of twist‐controlled electronic structure in anisotropic 2D materials, offering new pathways for designing reconfigurable quantum materials.

## Graphical abstract

Twist angle in stacked bilayer ReS_2_ can modulate 30% of interlayer coupling and the whole range of exciton energy between 1L and 2L ReS_2_.

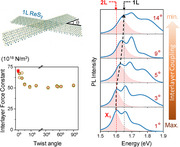

## 1 Introduction

ReS_2_ is a transition metal dichalcogenide (TMD) with a distorted 1T*'* phase and the intriguing characteristics of maintaining a direct bandgap and multiple exciton transitions originating from weak interlayer interaction. In‐plane anisotropic optical and electrical properties are observed in ReS_2_,^[^ ^1^, ^2^, ^3^, ^4^, ^5^, ^6^, ^7^ ^]^ which distinguishes it from the widely studied 2H type group‐VI TMDs. Recently, stacking two monolayers (1Ls) of a TMD with various twist angles has produced interesting correlated electronic phases and optical properties.^[^ ^8^, ^9^, ^10^, ^11^, ^12^, ^13^, ^14^ ^]^ Such a moiré superlattice can fundamentally modulate the interlayer interaction and lattice reconstruction, producing outstanding physical phenomena.^[^ ^8^, ^9^, ^12^ ^]^ For example, a moiré superlattice of twisted homo‐ or hetero‐bilayers composed of 1Ls of MoS_2_, MoSe_2_, WS_2_, and WSe_2_ displayed exotic phenomena such as bandgap tuning, circularly polarized interlayer excitons, multiple excitonic resonances, and long‐lived interlayer excitons.^[^ ^11^, ^12^, ^15^ ^]^

The interlayer interaction has been demonstrated in twisted bilayer (tBL) 2D‐TMDs to be particularly strong for small twist angles, which causes a significant lattice strain and lattice reconstruction, as confirmed using Raman spectroscopy, scanning tunneling microscopy (STM), and scanning transmission electron microscopy (STEM) techniques.^[^ ^8^, ^9^, ^10^ ^]^ tBL TMDs with small twist angles of less than 5° were found to have the characteristics of flat bands in their electronic structures, leading to interesting physical phenomena such as moiré exciton complexes, antiferromagnetic or ferromagnetic ordering, superconductivity, and the emergence of insulating phases.^[^ ^12^, ^13^, ^16^, ^17^, ^18^, ^19^, ^20^ ^]^ Furthermore, localized interlayer excitons, which are also called moiré excitons, were detected at an energy lower than the intralayer exciton energy in the photoluminescence (PL) spectra of tBL TMDs.^[^ ^18^, ^19^, ^20^ ^]^ By contrast, the energy of the indirect bandgap has been shown to display systematic modulation in response to the twist angle, while the intralayer exciton energy remained unchanged.^[^ ^11^, ^12^ ^]^ These studies of the dependence of the PL spectra on the twist angle of tBL have not been carried out for the low symmetry TMDs with 1T*'* lattice structure, such as ReS_2_ or ReSe_2_. Choi et al.^[^ ^21^ ^]^ identified the crystallographic orientation of tBL ReS_2_ using Raman spectroscopy, and Kong et al.^[^ ^22^ ^]^ showed the twist‐induced modulation of optical anisotropy in tBL ReS_2_, but the twist angle‐dependent bandgap, interlayer coupling strength, phonon renormalizations, and lattice reconstruction have not been studied.

In this work, we studied tBL ReS_2_ with various twist angles using PL and Raman spectroscopy, STEM imaging, and density functional theory (DFT) calculations. We found that the interlayer coupling of tBL ReS_2_ could be modulated by as much as 30% just by using a twist angle within ≈10°, and this modulated interlayer coupling had a direct effect on the bandgap energy, which was tuned over a range of 40 meV. This value was comparable to the difference in exciton energies between 1L ReS_2_ and intrinsic 2L ReS_2_. Our work provided an intriguing means to continuously modulate the optical properties of 2L ReS_2_.

## 2 Results and Discussion

### 2.1 Dependence of PL and Raman Spectra on Twist Angle

**Figure 1:**
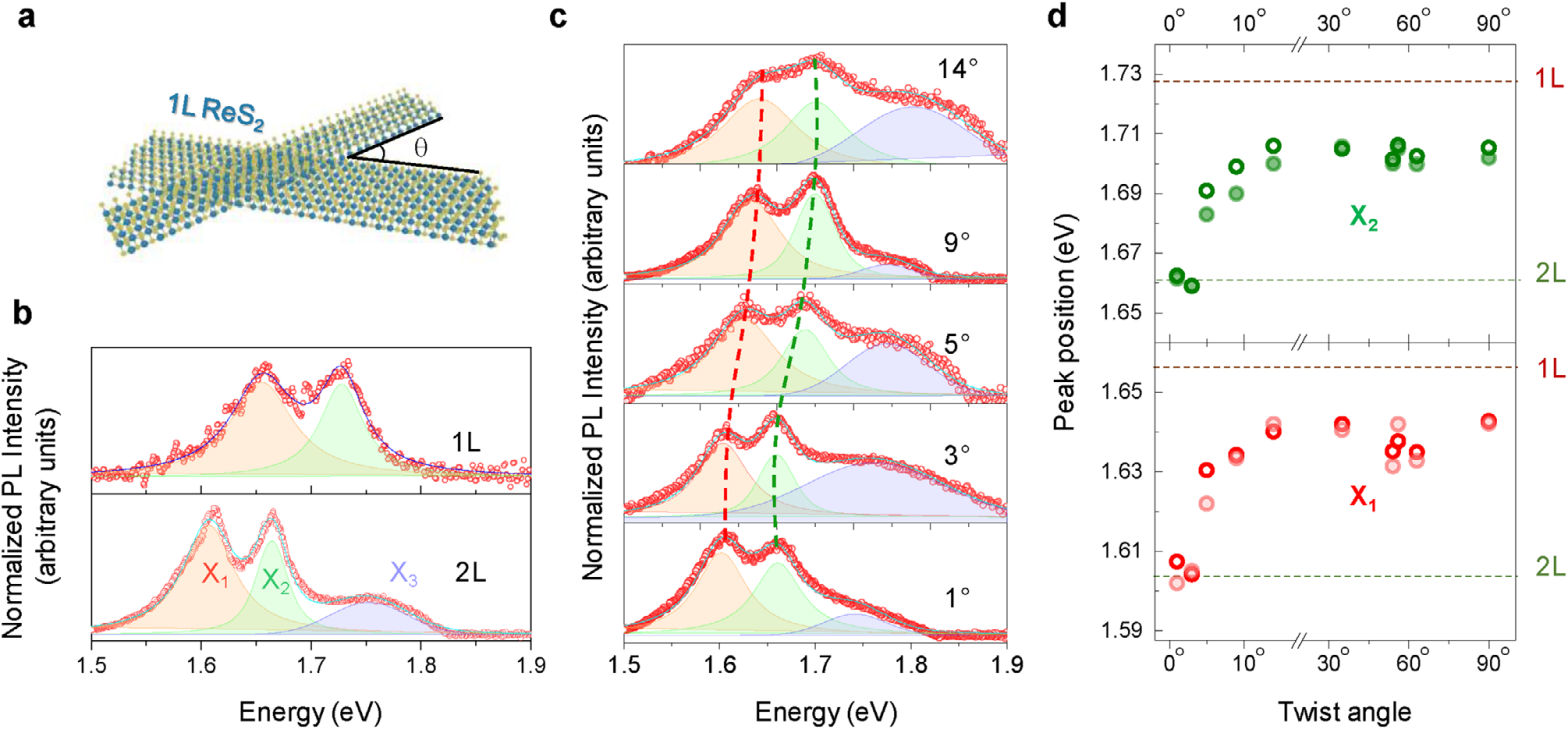
Modulation of exciton energies in the twisted bilayer (tBL) ReS_2_. a) Schematic of formation of tBL ReS_2_ structure using two monolayers (1Ls). b) PL spectra of the exfoliated intrinsic 1L and 2L ReS_2_ show the presence of three exciton peaks (X_1_, X_2_, and X_3_) through spectral fitting. c) PL spectra of tBL ReS_2_ with several selected twist angles. d) Peak position of the X_1_ and X_2_ peaks versus twist angle, where dotted lines indicate the peak positions of the exciton peaks from intrinsic 1L and bilayer ReS_2_. The different shades of data points represent the two different sets of measurement data. All the PL spectra were obtained at 3 K.

We prepared tBL ReS_2_ samples with various twist angles using a dry transfer method (see Experimental Section for the details of the sample preparation). The schematic shown in **Figure** **1**a represents the tBL ReS_2_ formed using two 1Ls of ReS_2_. Optical microscope views of 10 examples of the stacked tBL ReS_2_ with various twist angles are provided in Figure S1 (Supporting Information). We defined the twist angle as the angle between the in‐plane orientation (direction of b‐axes) of the top and bottom 1L ReS_2_ layers, where the b‐axes were identified as the long side of the monolayer (1L) ReS_2_ flake. Such identification of b‐axis is normally valid because of the preferred direction of mechanical cleavage in 1T*'* TMDs^[^ ^2^, ^23^ ^]^ as we confirmed such validity by using polarized Raman spectroscopy (example of identifying the crystal axis using Raman spectroscopy and experimental details are provided in Figure S2, Supporting Information). We didn't distinguish the out‐of‐plane orientation (c‐axis) of 1L ReS_2_,^[^ ^21^, ^22^ ^]^ thus only the twist range of 0°− 90° is defined in our measurements. PL spectra of the intrinsic (as‐exfoliated) 1L and 2L ReS_2_ are shown in Figure 1b. The PL spectra were collected at 3 K to easily distinguish the exciton peaks based on their narrow spectral widths. Three exciton peaks (X_1_, X_2_, and X_3_), which indicated the direct band transition at the Γ point,^[^ ^1^, ^5^, ^6^, ^7^ ^]^ were identified using fitting deconvolution of the PL spectra, as indicated by the shaded sub‐peaks.

The peak positions of X_1_, X_2_, and X_3_ for the intrinsic 1L or 2L ReS_2_ were consistent with the previously reported results that the exciton energies of 1L were 60–70 meV higher than those of 2L ReS_2_.^[^ ^5^, ^6^, ^7^ ^]^ Figure 1c shows the deconvoluted PL spectra of the tBL ReS_2_ with twist angles of 1°, 3°, 5°, 9°, and 14°. Two sets of PL results for other twisted angles are provided in Figure S3 (Supporting Information). We noticed that the exciton peak energies of X_1_, X_2_, and X_3_ gradually increased with the twist angle. Figure 1d shows a plot of the peak positions versus twist angle for all the inspected samples with various twist angles for the X_1_, and X_2_ peaks, which confirms the tendency for the peak energy of the exciton peaks to increase with the twist angle (X_3_ peak also showed the similar increase of the energy with the twist angle as shown in Figure S4, Supporting Information). A more detailed examination shows two facts that should be noted. First, the exciton peak positions changed mostly within ≈10°, at large twist angles beyond which they stayed nearly constant within the deviation among the separate measurements. Second, the range of change was ≈40 meV, which was comparable to the difference in the exciton energies (dotted lines) of the intrinsic 1L and 2L ReS_2_. Based on the exciton peak positions, tBL ReS_2_ with a twist angle larger than 10° seems to closely mimic the PL response of 1L‐ReS_2_, suggesting that a larger twist angle causes a very weak interlayer interaction between the top and bottom layers of tBL ReS_2_. This type of distinct change in the bandgap energy with the twist angle is not observed in a tBL of a 2H TMD such as tBL MoS_2_.

**Figure 2:**
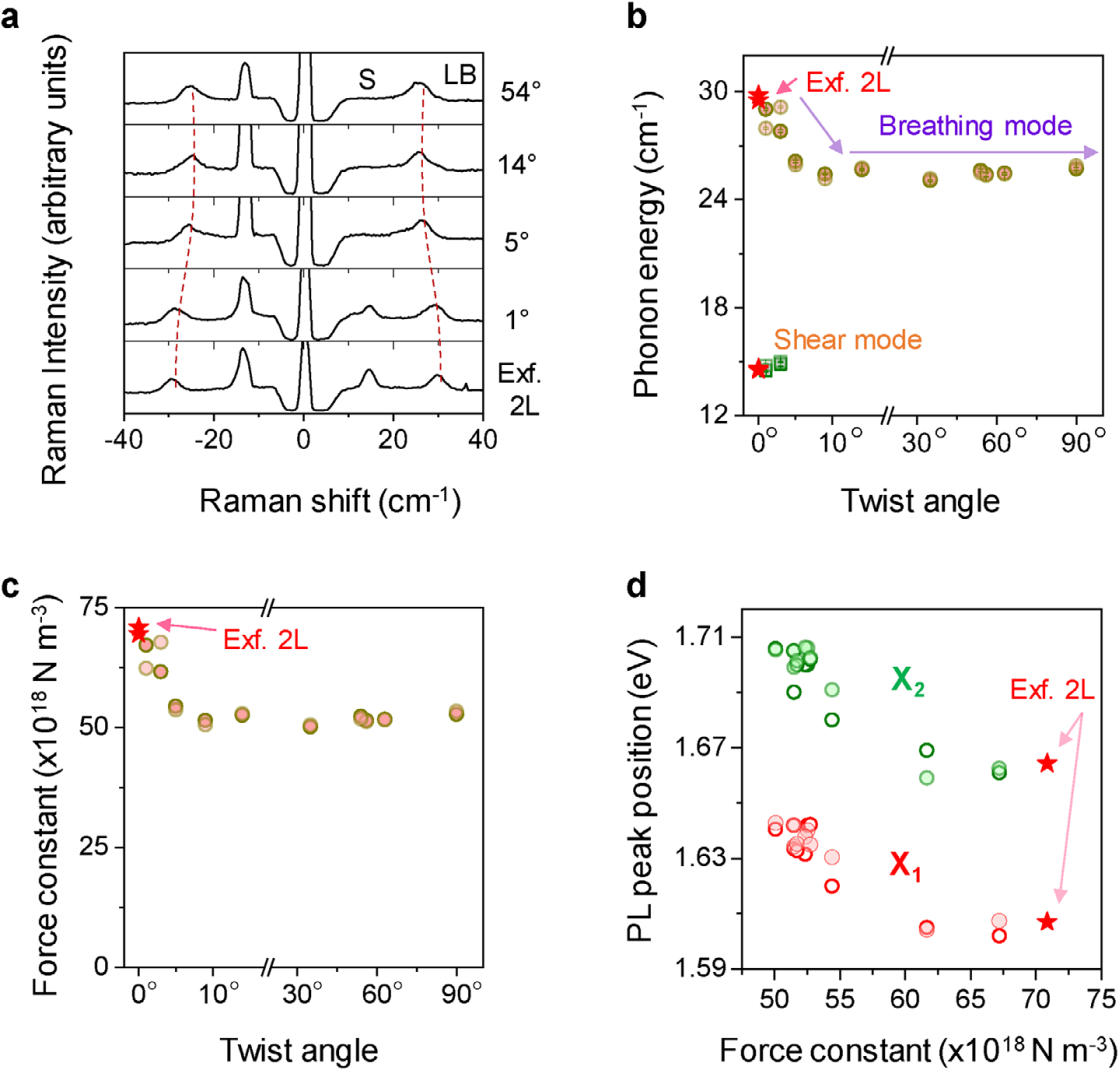
Modulation of low‐frequency Raman modes of tBL ReS_2_ with various twist angles. a) Low‐frequency Raman spectra of tBL ReS_2_ and exfoliated (Exf.) intrinsic bilayer (2L) ReS_2_ obtained with selected twist angles. Note that the shear modes in the anti‐Stokes Raman bands are obscured by the overlapping plasma line of the Ar‐ion laser (S: shear mode and LB: layer breathing mode). b) Plot of phonon energies versus twist angle. The phonon energies of the exfoliated intrinsic 2L ReS_2_ are shown for comparison (red stars). c) Plot of calculated force constant of interlayer coupling in tBL ReS_2_ versus twist angle. d) Correlation plots between the exciton (X_1_ and X_2_) energies determined in the PL spectra and the interlayer force constants for each twist angle investigated. The different shades of data points in Figure 2b–d represent the two different sets of measurement data.

We studied the dependency of low‐frequency Raman modes, such as the layer breathing (LB) and shear (S) modes of tBL ReS_2_ on the twist angle, with the Raman spectra shown in **Figure** **2**a. Raman spectra obtained with other twist angles are provided in Figure S5 (Supporting Information). The LB mode that appeared at ≈29 cm^−1^ for intrinsic 2L‐ReS_2_ is known to be a quantitative indication of the interlayer coupling strength in stacked 2L TMDs.^[^ ^8^, ^9^, ^24^ ^]^ The plot of the phonon modes versus twist angles in Figure 2b shows that a systematic softening of the LB mode with increasing twist angle up to ≈10° existed, beyond which it flattened out. This abrupt change in the LB modes with a 10° change in the twist angle was similar to the dependence of the exciton peak position on the twist angle of tBL ReS_2_ discussed above. The softening of the LB mode indicated the weakening of the interlayer coupling strength in tBL ReS_2_ as the twist angle increased. The absence of the shear mode at 15 cm^−1^ beyond a twist angle of 5° also suggested a significant reduction in interlayer coupling with increasing twist angle because this absence of a shear mode has been reported for other twisted TMD structures.^[^ ^8^, ^9^ ^]^ The force constant (*K*) of the interlayer coupling could be estimated based on the phonon energy of the LB mode (see the calculation details in the SI), and the results are shown in Figure 2c. We see that *K* decreased from 69.0 to 48.5 × 10^18^ N m^3^ as the twist angle became larger than 10°. This change (≈30%) in interlayer coupling as a result of the twist angle was much larger than the previously reported changes in stacked 2L‐MoS_2_^[^ ^25^ ^]^ and consistent with the observed large change in the exciton peak with the twist angle. Figure 2d shows the correlation plot between the PL peak positions of the X_1_ and X_2_ excitons and the interlayer force constants for each twist angle considered. The high degree of correlation confirmed that the observed modulation of exciton energy based on the twist angle was the result of modulated interlayer coupling.

**Figure 3:**
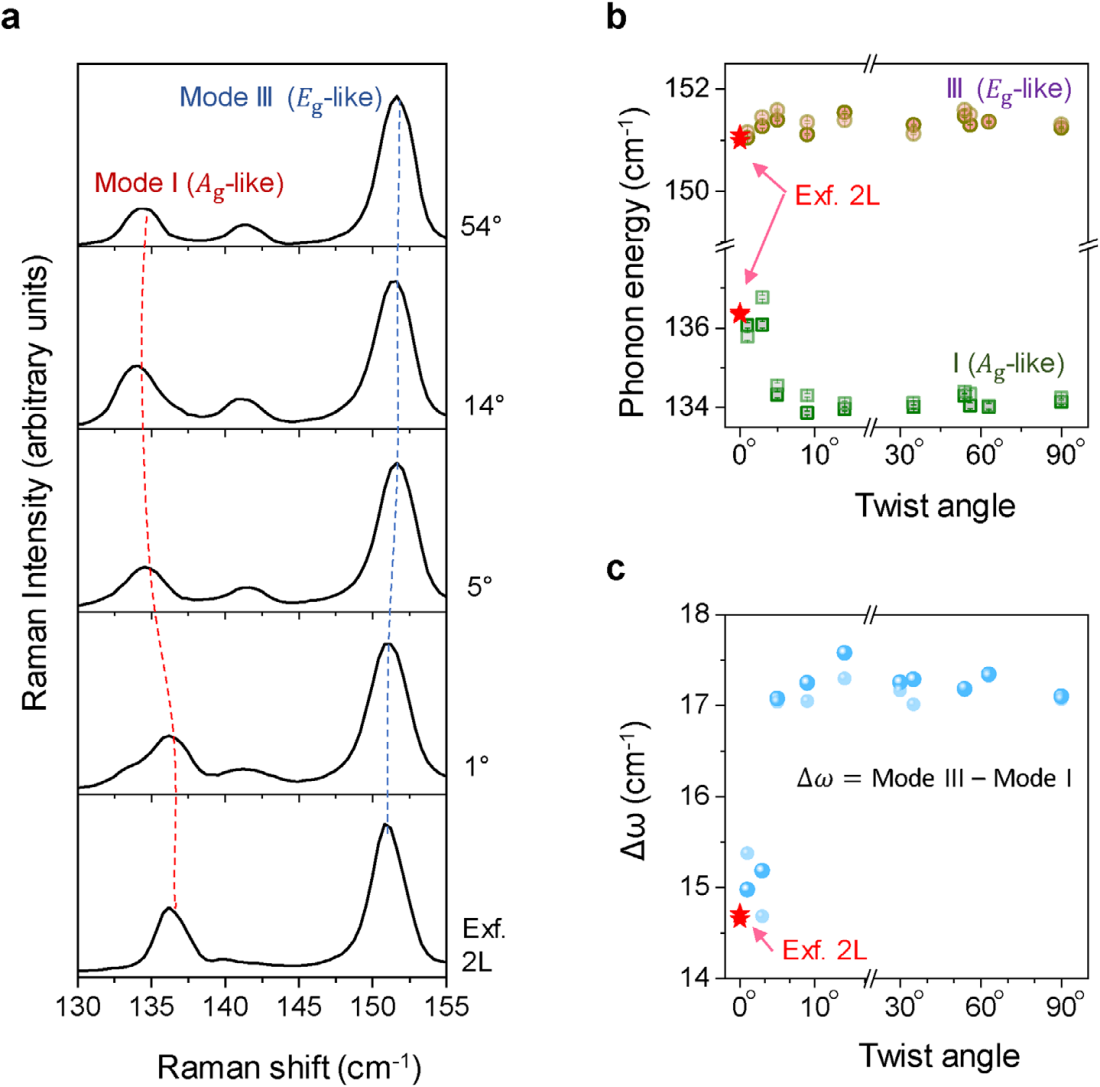
Modulation of high‐frequency Raman modes of tBL ReS_2_ with various twist angles. a) Raman spectra of tBL ReS_2_ with selected twist angles and exfoliated intrinsic 2L ReS_2_. A_g_‐like (Mode I) and E_g_‐like (Mode III) Raman modes are indicated in the spectra. Dashed lines are guides for the eyes to show the peak shifts. b) Plot of peak positions of the A_g_‐like and E_g_‐like Raman modes versus twist angle. The red stars in the figure indicate the corresponding Raman frequency of the exfoliated intrinsic bi‐layer ReS_2_. c) Plot of frequency difference between E_g_‐like and A_g_‐like Raman modes versus twist angle. The different shades of data points represent the two different sets of measurement data.

We also measured the high‐energy region of the Raman modes of tBL ReS_2_ with various twist angles. ReS_2_ is a low‐symmetry crystal with 18 irreducible in‐plane‐like and out‐of‐plane‐like Raman modes.^[^ ^26^, ^27^, ^28^, ^29^ ^]^ In the spectral region between 130 and 240 cm^−1^, six optical phonon modes were observed in the Raman spectra of tBL ReS_2_ with different twist angles, corresponding to the out‐of‐plane *A*_g_‐like (ω_1_, ω_2_) and in‐plane *E*_g_‐like (ω_3_, ω_4_, ω_5_, ω_6_) modes (Figure S6, Supporting Information). **Figure** **3**a shows the twist angle dependent *A*_g_‐like and *E*_g_‐like Re─Re stretching Raman modes, as indicated by ω_1_ (mode I) and ω_3_ (mode III), respectively, where the Raman spectrum of the intrinsic 2L ReS_2_ is also shown for comparison. A plot of the peak positions of mode I and mode III as a function of the twist angle is shown in Figure 3b, where the energy of the *E*_g_‐like mode slightly increases by 0.5  ±  0.42 cm^−1^ with an increase in the twist angle from 1° to 5° and then saturates for larger twist angles. The energy of the *A*_g_‐like mode decreases by 2.5 cm^−1^ as the twist angle changes from 0° to 5° and then remains almost the same for larger twist angles. A plot of the energy difference between the *E*_g_‐like and *A*_g_‐like modes versus twist angles shown in Figure 3c indicate the strong correlation between the phonon energy and twist angle more distinctly. This resembles the layer breathing Raman modes, confirming the gradual weakening of the interlayer coupling of 2L‐ReS_2_ with increasing twist angle within ≈10°. We also plotted each of the *A*_g_‐like and *E*_g_‐like phonon energies versus twist angles for other Raman modes, as shown in Figure S7 (Supporting Information). Because of their distinct origins at each mode, all of the *A*_g_‐like modes were softened, and the *E*_g_‐like modes were stiffened, following the same general trends shown for the mode I and mode III, but with different values.

The softening of the *A*_g_‐like mode with increasing twist angle was strongly related to the weakening of the interlayer coupling strength with an increase in the interlayer distance between two adjacent layers. For example, the decreased frequency in the out‐of‐plane *A*_1g_ the mode in both twisted 2L‐MoS_2_ and 2L‐WS_2_ was found to be due to the increased interlayer distance.^[^ ^25^, ^30^, ^31^ ^]^ In particular, the low‐energy breathing mode was more influenced by the interlayer distance and coupling related to the twist angle, as previously observed in twisted 2L‐MoS_2_.^[^ ^25^ ^]^ Similarly, in our case, the low‐energy breathing mode decreased by ≈4.5 cm^−1^ with an increase in the twist angle toward 10°, which was much larger than the 2.5 cm^−1^ decrease observed in the *A*_g_‐like mode I (Figures S6 and S7, Supporting Information). Our Raman results suggested that the interlayer distance gradually increased with the twist angle up to 10° and was nearly constant for larger twist angles. We used the atomic force microscope (AFM) to measure the thicknesses of our tBL ReS_2_ samples and found that height of top layer from the bottom layer was consistently higher at large twist angles than at small angle less than 10° (AFM images and cross‐sectional height profiles for selected tBL samples are provided in Figure S8, Supporting Information), which supports the results of Raman measurements that interlayer coupling gradually decreased with the twist angle. Electrical transport on selected angles of tBL ReS_2_ was also measured. As the results and analysis are provided in Figure S9 (Supporting Information), the estimated field‐effect mobility of tBL ReS_2_ with a twist angle of 1° was close to the value of intrinsic 2L ReS_2_, while the sample of 6° twist angle showed a substantially lower value. This result of relatively higher mobility at larger twist angle is consistent with the observation of weak interlayer coupling of tBL ReS_2_ at large twist angle which was shown in our PL, Raman, and AFM studies.

Strong dependence of interlayer interaction occurring at small twist angle of tBL ReS_2_ observed in our PL and Raman study is consistent with previous works, which showed the large shift of LB modes in tBL MoS_2_ and MoSe_2_ at small twist angle within 10°.^[^ ^8^, ^9^, ^32^ ^]^ The explanation for enhanced moiré effects at small twist angles is that the period of the moiré superlattice is longer, causing stronger interlayer hybridization and stronger periodic potential modulation that electrons and excitons experience.^[^ ^8^, ^9^, ^12^ ^]^ ReS_2_ has an intrinsic low symmetry of 1T*'* structure along with strong in‐plane anisotropy, which may be the reason for the observed greater modulation of interlayer coupling and the bandgap with the twist angle.

### 2.2 Strain Analysis by STEM

Considering the fact that the Raman results revealed an increase in the stiffening effect along the in‐plane directions in the bilayers, along with increasing softening for the interlayer coupling with an increase in the twist angle, we expected the relative strain between the layers along the in‐plane direction to be different from that of a non‐strained ReS_2_ bilayer. We observed the plane‐view atomic structures of the ReS_2_ bilayers relatively twisted by 0°, 3°, and 43° using high‐angle annular dark field (HAADF) STEM imaging to examine the plausible in‐plane strain effect due to the characteristic in‐plane stiffening coupling in the twisted ReS_2_ bilayer.^[^ ^26^, ^33^, ^34^ ^]^ With non‐strained atomic models of the ReS_2_ bilayers with the same twist angles, we simulated HAADF STEM images as reference images to evaluate the in‐plane strain effect of the experimental ReS_2_ bilayers in relation to the twist angle. First, before estimating the lattice strain state of the experimental ReS_2_ bilayers by comparing the reference simulated image, validating whether the HAADF STEM image of a 1T*'* ReS_2_ monolayer was a good match to the counterpart simulated image that was generated using the known atomic structure of 1T' ReS_2_ was necessary. Thus, we compared the experimental HAADF STEM image of the 1T' ReS_2_ monolayer to the simulated image (Figure S10). The result showed that the projected atomic distances between Re atoms measured from the simulated HAADF STEM image had a one‐to‐one correspondence with those measured from the experimental one, confirming that the monolayer ReS_2_ was identical to the non‐strained 1T' ReS_2_ structure.

**Figure 4:**
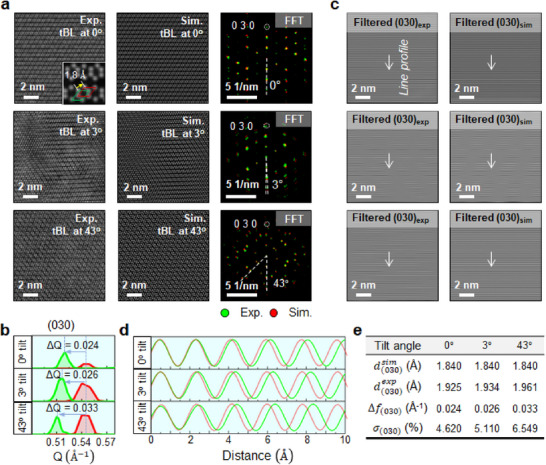
Plane‐view atomic structure observations of the exfoliated 1T*'* ReS_2_ bilayer without twist and twist 1T*'* ReS_2_ bilayers with different twist angles of 3° and 43°. a) Experimental (left column) and simulated (middle column) HAADF STEM images of the three ReS_2_ bilayers, along with overlapping FFT patterns of the experimental and simulated HAADF STEM images (right column). The FFT patterns in green and red indicate the FFT results for the experimental and simulated HAADF STEM images, respectively. Note that one layer of the exfoliated 1T’ ReS_2_ bilayer was observed to relatively slide to the other layer by a spacing (1.8 Å) corresponding to the (300) plane (see inset). b) Comparison of (030) spots in the FFT patterns of the experimental and simulated HAADF STEM images of the three ReS_2_ samples. c) Bragg‐filtered images obtained from the FFT patterns of the experimental and simulated HAADF STEM images by choosing (030) spots. d) Comparison of line profiles (marked in c) across the (030) planes between the experimental and simulated images for the three ReS_2_ samples. e) List of the (030) planar spacing values measured from the experimental images and calculated strains for the (030) plane of the three ReS_2_ samples compared to the simulated image of the non‐strained ReS_2_.

The leftmost column of **Figure** **4**a shows plane‐view atomic resolution HAADF STEM images of the exfoliated 1T*'* ReS_2_ bilayer without a twist and the tBL 1T*'* ReS_2_ samples with different twist angles of 3° and 43°. The simulated images of the corresponding bilayer models with the same twist angles are displayed in the middle column of Figure 4a. The simulated images corresponded very well with their experimental counterparts for all three samples, as shown. Intriguingly, we observed characteristic layer sliding in the exfoliated ReS_2_ bilayer by a distance of 1.8 Å along the [100] direction. This sliding distance corresponded to the (300) planar spacing (see inset in the simulated image). This result suggested that the interlayer interaction was different from that in the case of the ReS_2_ bilayer with A‐A stacking. We compared fast Fourier transform (FFT) patterns^[^ ^35^ ^]^ of the experimental HAADF STEM images with those of the simulated images to examine whether the lattices of the ReS_2_ bilayers were distorted due to the increase of the in‐plane stiffening effect with the twist angle, as shown in the rightmost column of Figure 4a. Remarkably, the overall FFT patterns (green) of the experimental HAADF STEM images appeared to be shrunken compared to the FFT patterns (red) of the simulated images. These results indicated that all the lattices were somewhat expanded in the in‐plane directions compared to the non‐strained state of the 1T*'* ReS_2_ lattice. A comparison of the (030) spots in the FFT patterns calculated from the experimental and simulated HAADF STEM images confirmed that the magnitude of the lattice expansion of the bilayer samples increased with the twist angle (Figure 4b). We obtained Bragg‐filtered images by selecting the (030) spots in the FFT patterns to evaluate the lattice strain occurring along the [010] direction, as shown in Figure 4c, and then compared the intensity profiles obtained across the line fringe patterns (Figure 4d). The peak‐to‐peak distances measured in those profiles corresponded to the planar spacing of the (030) plane. The results showed that the planar spacing ($d_{\left(030\right)}^{exp}$) of the (030) plane gradually expanded as the twist angle increased, in comparison to the value ($d_{\left(030\right)}^{sim}$) of the non‐strained state. The tensile strain applied in the array of the (030) planes in the tBL ReS_2_ was measured to increase from 4.62 to 6.55% when the twist angle increased from 0° to 43° (Figure 4e). The exfoliated ReS_2_ bilayer without a twist even showed a noticeable tensile strain in the in‐plane directions, which suggested the presence of significant interlayer coupling between the layers due to the relative layer sliding. Given that a tensile strain would reduce the bandgap energy, the results of our strain analysis implied that the observed strain phenomenon was one of the ramifications of the interlayer coupling caused by the layer sliding and twist operations. Thus, we presumed that the observed lattice distortions were not the main reason for the increasing bandgap energy of the stacked tBL ReS_2_ shown in Figure 1.

### 2.3 Calculated Band Structures

To investigate the evolution of the bandgap of tBL ReS_2_ with increasing twist angle, we performed DFT calculations as described later in the experimental section. The unit cells of the moiré superlattice were generated by first relaxing an untwisted bilayer of ReS_2_, resulting in an interlayer Re─Re distance of 5.85 Å and a hexagonal unit cell with lattice constant a = 6.44 Å. Specific twist angles were chosen to minimize the size of the moiré superlattices, identical to the twist angles typically chosen to model tBLs of 2H‐TMDs.^[^ ^16^ ^]^ While the size of the moiré superlattice doesn't exactly depend on the twist angle, there is a lower limit at specific angles that increases significantly with decreasing twist angle,^[^ ^36^ ^]^ therefore the minimum modeled twist angle was 7.34°.

**Figure 5:**
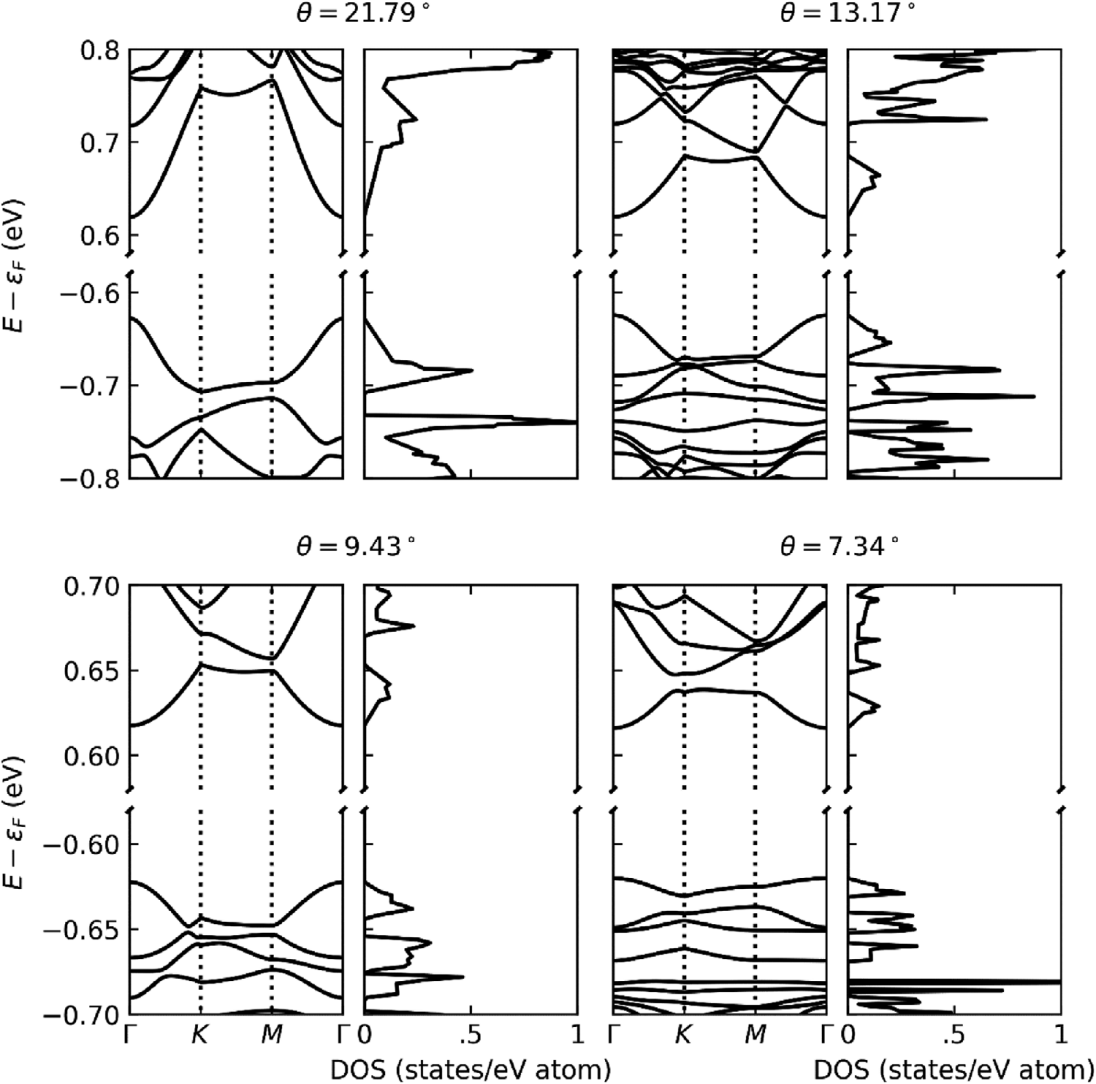
DFT calculation of electronic band structures and their associated DOS of tBL ReS_2_ calculated at twist angles of 21.79°, 13.17°, 9.43°, and 7.34°. It is worth noting that the energy scale plotted for θ = 9.43° and 7.34° is half that plotted for θ = 21.79° and 13.17°.

**Figure 6:**
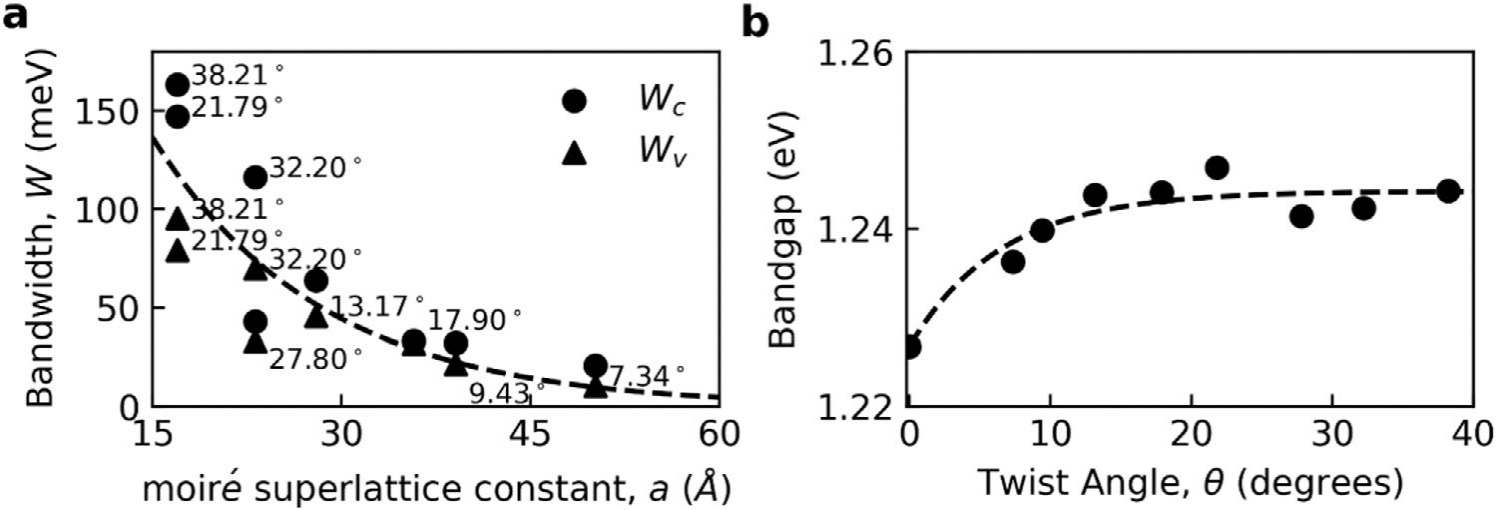
a) Conduction and valence bandwidth as a function of the size of the moiré superlattices, which in general increases at smaller twist angles. The dashed line is a guideline to show that both bands become significantly flatter at lower twist angles. b) Direct bandgap of tBL ReS_2_ between the conduction and valence bands at the Γ point as the angle between the layers is modified, the dashed line is a guideline to show leveling‐off of bandgap when θ >10°.

The calculated band structures of the tBL ReS_2_ in **Figure** **5** show increasingly flat band characteristics with decreasing twist angle, similar to the results reported for tBLs of 2H‐TMDs^[^ ^14^, ^16^ ^]^ (calculated band structures of the untwisted bilayer ReS_2_ is provided in the Figure S11 (Supporting Information) for comparison, which is distinctly different than the result of twisted bilayers). Their origin has been attributed to inhomogeneous interlayer hybridization in the moiré superlattice.^[^ ^12^, ^14^, ^16^, ^19^, ^37^ ^]^ As the twist angle of tBL ReS_2_ is reduced, going from 21.79°, 13.17°, 9.43°, and finally 7.34°, the size of the hexagonal moiré superlattice increases its side length from a = 16.98, 27.98, 39.04, and finally 50.13 Å. While the general features of the bands and density of states (DOS) remain the same as the twist angle is reduced, the periodicity of the bands is decreased, inversely proportional to the increased periodicity of the moiré superlattice in real space. This results in the bands becoming flatter with decreasing twist angle, and the relatively low‐energy (high‐energy) K and M symmetry points of the valence (conduction) band begin to approach that of the relatively high‐energy (low‐energy) Γ symmetry point, where the direct bandgap appears. Interestingly, decreasing the twist angle of tBL ReS_2_ results in the flattening of both the valence and conduction bands as opposed to only flattening the valence band of tBLs of 2H‐TMDs and the conduction band remaining parabolic.^[^ ^16^ ^]^ To quantify this, the bandwidth of the flat valence and conduction bands can be defined^[^ ^38^ ^]^ as W_v_ = E_v_(Γ) – E_v_(K) and W_c_ = E_c_(M) – E_c_(Γ) respectively, where E_v_ and E_c_ are the energies of the valence and conduction bands. It can be seen in **Figure** **6**a that as the twist angle is decreased from 38.21° to 9.43°, the bandwidth of the valence and conduction bands dramatically decreases from 160 and 95 meV to 30 and 20 meV. For comparison, similar DFT calculations for tBLs of 2H‐TMDs such as MoS_2_ with a twist angle of 7.34° have a valence bandwidth of 60 meV and a conduction bandwidth of 150 meV.^[^ ^16^ ^]^

The appearance of flat bands in both the valence and conduction bands could have profound effects on the electronic transport in tBL ReS_2_. The flattening of both bands would lead to a reduction in the Fermi velocity of both excited electrons in the conduction band and holes in the valence band, approaching zero as the bands become perfectly flat. The flattening of the bands also reduces the overall number of states available to electrons in the conduction band and holes in the valence band. This can be seen as a reduction in the DOS by ≈50% as the twist angle is decreased from θ = 21.79° to 13.17°. Additionally, the narrowing of the DOS from θ = 13.17° to 9.43° reduces the total number of states available to the bands without decreasing the magnitude of the DOS. This reduction in the number of available states would lead to longer‐lived electrons and holes in the conduction and valence bands as they have fewer available states to scatter to. This would lead to longer‐lived excitons than in 2H‐TMDs, where parabolic conduction bands provide plenty of states for electrons to scatter to. Excitons in tBL ReS_2_ become increasingly long‐lived and immobile as the twist angle is reduced and the bands become increasingly flat. Such correlation between the formation and resultant phenomena of intriguing quantum transport could be quantitatively explored by further studies.

In Figure 6b, the bandgap energies estimated from the calculated band structures are plotted as a function of the twist angle of tBL ReS_2_. Noticeably, the bandgap showed a gradual increase with the twist angle, and this tendency was similar to that of the gradual decrease in the interlayer coupling with an increasing twist angle under 10°, as discussed using the results of PL and Raman spectroscopy. Our observation was also consistent with the general tendency that the interlayer coupling and bandgap of tBL TMD decrease with an increasing number of layers.^[^ ^5^, ^7^, ^11^ ^]^ One interesting perspective is that a similar trend of modified interlayer coupling and bandgap energy with twist angle could be observed in tBL ReSe_2_, because ReSe_2_ has the same 1T*'* crystal structure of strong in‐plane anisotropy as ReS_2_. The bandgap of ReSe_2_ is smaller than ReS_2_,^[^ ^4^, ^5^ ^]^ which can be related to the interlayer coupling strength by noting Se has valence electrons in higher‐energy orbitals than S, resulting in the wavefunctions of the *p_z_*‐orbitals overlapping more with those of adjacent layers to create stronger bonds. This may have competing effects on the interlayer coupling and on how strong the dependence of interlayer coupling or bandgap on twist angle would be for tBL ReSe_2_.

## 3 Conclusion

In conclusion, we demonstrated that the interlayer interaction tBL ReS_2_ can be modulated by ≈30% just by the twist angles, where the strongest interaction occurred with a small angle of less than 10°. The energy of the main exciton peaks changed as a function of the twist angle in correlation with the interlayer interaction between the stacking layers. The STEM results indicated that the lattice strain manifested in the tBL ReS_2_ samples could be attributed to the change in interlayer coupling caused by the layer sliding and twist operations. Our work provides an effective and versatile means to engineer the band structure and electronic properties of 1T*'*‐phase ReS_2_ for further application in optoelectronic devices. Possible applications for the adjustable bandgap of tBL ReS_2_ are tunable near infra‐red light sources and photodetectors without the need for structural modification by electrical gating or strain. Moreover, strong modulation of band structure by twist angle along with the intrinsic low‐symmetry crystal structure naturally suggests that tBL ReS_2_ provides an enhanced platform to study twistronics quantum phenomena, including moiré‐confined excitons and correlated electronic states.

## 4 Experimental Section

### Preparation of Samples

The Scotch tape method was first used to exfoliate bulk 1T*'*‐ReS_2_ crystals (2D Semiconductor Supplies Corp.) onto a polydimethylsiloxane (PDMS) film adhered to a glass slide to make handling easier. Different monolayers were examined using an optical microscope and then stacked with various twist angles on the SiO_2_/Si substrate using the dry transfer technique.^[^ ^39^ ^]^ The sample was cleaned by heating it for 2 h in an Ar gas environment to remove the residue caused by the exfoliation processes and to make firm contact between the two stacked layers prior to the measurements.

### PL and Raman Spectral Measurements

Confocal PL spectral measurements were performed on a lab‐made confocal microscope system.^[^ ^40^ ^]^ An Ar ion laser with an excitation wavelength of 514.5 nm was used at a laser power of 500 µW. For low‐temperature (3 K) PL measurements, an objective lens with a numerical aperture of 0.6 was used to illuminate the samples in a vacuum chamber (Montana Instruments Cryostation s50).^[^ ^41^ ^]^ The collected light was guided to a 50 cm‐long monochromator equipped with a cooled charge‐coupled device through an optical fiber with a core diameter of 100 µm; this acted as a confocal detection pinhole. Diffraction gratings with 150 grooves mm^−1^ were used for PL. For the low‐ and high‐energy Raman study, a bandpass filter and three‐notch filters with narrow bandwidths were employed in the confocal microscope system. A diffraction grating with 2400 grooves mm^−1^ was used for the Raman spectroscopy.^[^ ^42^ ^]^ Raman spectra were acquired using a parallel polarization configuration, where both the incident and scattered polarization directions were aligned along the long axis (b‐axis) of the bottom layer in each tBL sample. For polarized Raman measurements, the incident laser polarization was rotated relative to the long axis of the bottom ReS_2_ layer, while the scattered polarization remained parallel to this axis.

### STEM Characterization

The in‐plane atomic structures of the ReS_2_ bilayer samples were imaged in a HAADF STEM imaging mode using aberration‐corrected STEM (ARM200CF, JEOL Ltd.) operating at 80 kV. The collection angle of the HAADF detector and the convergence angle of the scanning electron probe were set to ≈70 − 175 mrad and 23 mrad, respectively. The electron dose rate was limited to ≈6  ×  10^6^ e − s^−1^nm^−2^ to avoid electron beam‐induced damage (or distortion) to the samples, which was optimized for observing the ReS_2_ monolayer in the previous study.^[^ ^26^ ^]^ The noise backgrounds in the experimental images of the ReS_2_ bilayer samples were removed by the Wiener filtering process using commercial software (HREM Filter Pro, HREM Research Ltd.). Simulations of the electron diffraction and STEM images of the 1T*'* ReS_2_ bilayer models were performed with experimental microscope observation parameters using the JEMS software package and QSTEM software package,^[^ ^43^ ^]^ respectively. Quantitative atomic position and bond length measurements for the experimental and simulated STEM images of the ReS_2_ monolayer were conducted using MATLAB‐based home‐built software.

### Electrical Transport

The channels of twisted bilayer ReS_2_ were patterned using a reactive ion etcher and electron beam lithography, which was followed by metallization using Cr and Au. Three devices were fabricated with bilayer ReS_2_ at different twist angles between the top and bottom layers, i.e., 0°, 1°, and 6°, at temperatures of 300, 200, and 100 K.

### DFT Calculation

The electronic structure calculations were carried out using a linear combination of atomic orbitals (LCAO), as implemented in QuantumATK,^[^ ^44^ ^]^ where the Kohn–Sham (KS) Hamiltonian was represented on the basis of PseudoDojo norm‐conserving pseudopotentials,^[^ ^45^ ^]^ using a density mesh cut‐off of 1225 eV and an energy convergence of 10^−5^ eV, along with a 5 × 5 × 1 k‐point mesh for the untwisted bilayer, 2 × 2 × 1 mesh for the smaller supercells (twist angles of 21.79°, 27.8°, 32.2°, and 38.21°), and 1 × 1 × 1 mesh for the larger supercells (twist angles of 7.34°, 9.43°, 13.17°, and 17.9°). The electron–core interactions were described with the Perdew–Burke–Ernzerhof (PBE) parameterization of the generalized gradient approximation (GGA),^[^ ^46^ ^]^ and the Grimme DFT‐D3 dispersion correction^[^ ^47^ ^]^ was included. The untwisted bilayer was minimized with a force convergence criterion of 10^−2^ eV Å^−1^ and maximum stress of 0.1 GPa. The twisted structures were constructed using the Interface Builder tool of Quantum ATK by setting a 0% lattice mismatch.

## Conflict of Interest

The authors declare no conflict of interest.

## Author Contributions

K.P.D., T.T.T., T.L., W.C., and S.F.P. contributed equally to this work. K.P.D. conceived the project, analyzed the data, and wrote the manuscript. T.T.T. prepared the samples. T.T.T. and K.P.D. performed optical measurements and analyzed the data. T.L. and D.L. performed optical measurements and analyzed the data. W.C. performed the STEM measurements and analyzed the data. J.M.M.T., S.F.P., and M.A.M. performed theoretical calculations and wrote the manuscript. V.K.D. and J.H.K. helped to prepare the samples. J.B. and S.C.L. measured the electrical transport properties and wrote the manuscript. Y.M.K. and H.R. analyzed the data and wrote the manuscript. J.K. conceived and supervised the project, analyzed the data, and wrote the manuscript.

## Supporting information



Supporting Information

## Data Availability

The data that support the findings of this study are available from the corresponding author upon reasonable request.
