# Supplementary material for: Giant Modulation of Interlayer Coupling in Twisted Bilayer ReS2
Source: Adv Sci (Weinh). 2025 Apr 25;12(23):2500411. doi: 10.1002/advs.202500411 (PMC12199411; doi:10.1002/advs.202500411)
Supplement: Supplementary file 1 — Supporting Information [file ADVS-12-2500411-s001.docx]

Supporting Information

**Giant Modulation of Interlayer Coupling in Twisted Bilayer ReS_2_**

*Krishna P. Dhakal^1^, Trang Thu Tran^1^, Taegeon Lee^2^, Wooseon Choi^1^, Sean F. Peterson^3^, Juan M Marmolejo‐Tejada^4,5^, Jaeuk Bahng^6^, Daekwon Lee^2^, Vu Khac Dat^1^, Ji-Hee Kim^7^, Seong Chu Lim^1,6^, Martín A Mosquera^4^, Young-Min Kim^1,8,^*, Heesuk Rho^2^*, Jeongyong Kim^1,^**

K. P. Dhakal, T. T. Tran, W. Choi, V. K. Dat, S. C. Lim, Y.-M. Kim, J. Kim

^1^Department of Energy Science, Sungkyunkwan University, Suwon, 16419, Republic of Korea.

^2^Department of Physics, Research Institute for Materials and Energy Sciences, Jeonbuk National University, Jeonju, 54896, Republic of Korea.

^3^Department of Physics, Montana State University Bozeman, MT 59717, USA.

^4^Department of Chemistry and Biochemistry, Montana State University, Bozeman, MT 59717, USA.

^5^Efficient Power Conversion Corp., El Segundo, CA 90245, USA.

^6^Department of Smart Fabrication Technology, Sungkyunkwan University, Suwon, 16419, Republic of Korea.

^7^Department of Physics, Pusan National University, Busan, 46241, Republic of Korea.

^8^Center for 2D Quantum Heterostructures, Institute for Basic Science (IBS), Suwon, 16419, Republic of Korea.

E-mail: [youngmk@skku.edu](mailto:youngmk@skku.edu); [rho@jbnu.ac.kr](mailto:rho@jbnu.ac.kr); [j.kim@skku.edu](mailto:j.kim@skku.edu)


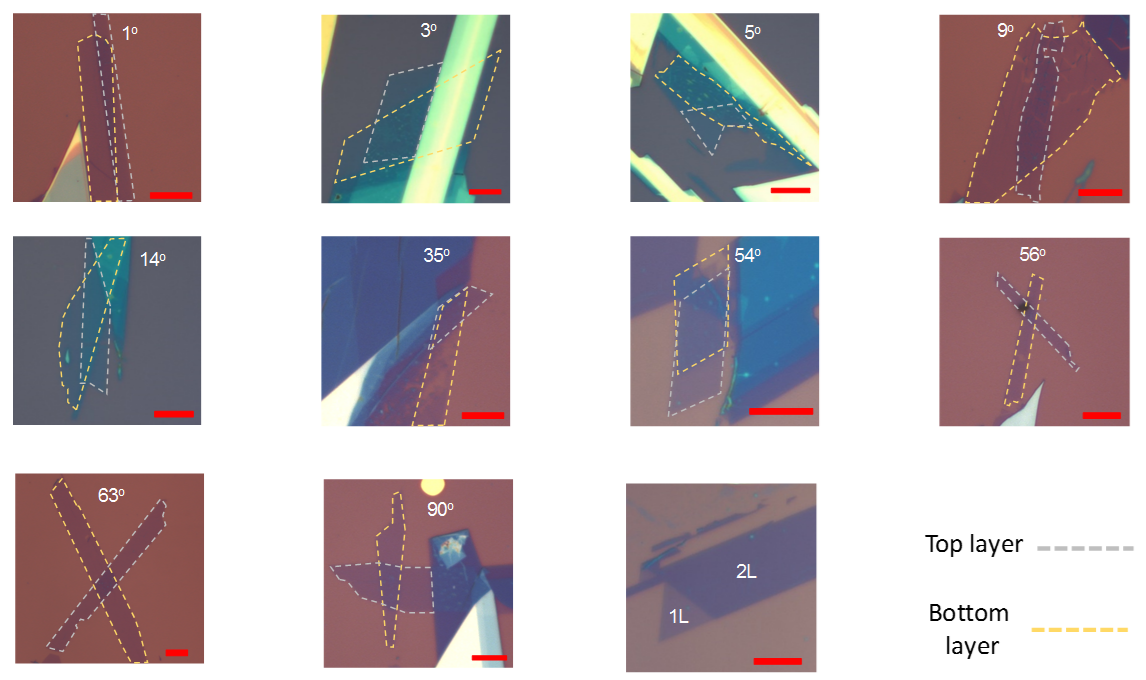


**Figure S1.** Optical microscope views of all tBL ReS_2_ samples studied. The regions of the top and bottom 1L-ReS_2_ flakes are highlighted by dashed gray and yellow lines, respectively. All scale bars are 10 µm.

**Identification of In-plane crystal orientation**

Specifically, Raman mode V (212 cm^-1^) exhibits maximum intensity at φ = 0° and minimum intensity at φ = 90° relative to the b-axis of ReS_2_.^[1–3]^ This characteristic allows the b-axis of ReS_2_ to be identified via polarized Raman scattering. To determine the crystallographic orientation of ReS_2_ in our study, we performed polarized Raman measurements on a ReS_2_ sample. During these measurements, the incident laser polarization was rotated relative to the long axis of the ReS_2_ layer while keeping the scattered polarization parallel to this axis. The inset in **Figure** **S2a** shows an optical microscope image of the bilayer ReS_2_, illustrating the experimental geometry of the polarized Raman scattering setup, including the incident (dotted line) and scattered (dashed line) polarization directions and the polarization angle φ. Figure S2a presents two representative polarized Raman spectra for parallel (φ = 0°) and perpendicular (φ = 90°) polarization configurations. Figure S2b displays a polar plot of the angle-resolved intensities of Raman mode V, revealing a two-lobed profile with a maximum at φ = 0°. This confirms that the long axis of the sample corresponds to the b-axis of ReS_2_, consistent with previous reports.^[1–5]^


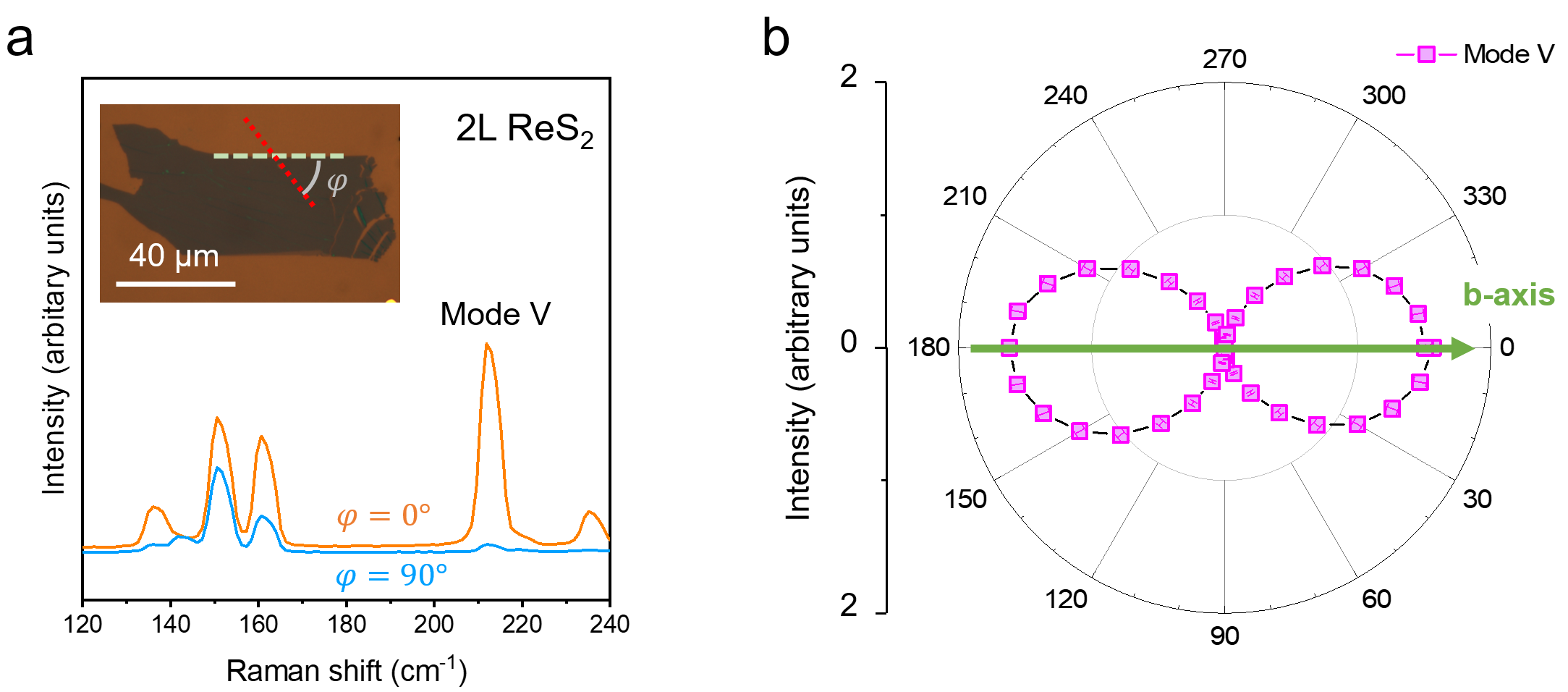


Figure S2. (a) Representative Raman spectra of 2L ReS_2_ obtained at polarization angles φ = 0° and φ = 90°. The inset shows an optical microscope image of the 2L ReS_2_, where the dashed line represents the polarization direction of the scattered light aligned with the sample’s long axis (b-axis), and the dotted line indicates the polarization direction of the incident laser light. (b) Polar plot of the intensity variation of Raman mode V as a function of polarization angle φ. The arrow denotes the b-axis of the 2L ReS_2_.

**Figure S3.** (a,b) PL spectra of the tBL ReS_2_ obtained at 3K for all twist angles measured. Vertical dashed lines and red arrows are guides for eyes to see the shift of the exciton peak with the twist angle. All the PL spectra were obtained at 3 K.

**Figure S4:** Peak position of the X_3_ peaks vs. twist angle, where dotted lines indicate the peak positions of the exciton peaks from intrinsic bilayer ReS_2_. The different shades of data points represent the two different sets of measurement data.

**Calculation of interlayer force constant**

Force constants $K_{S}$ and $K_{LB}$ for the shear and layer breathing modes, respectively, can be calculated using a linear chain model: $K_{S, LB}=\left( 2\pi^{2}c^{2} \right)\mu\omega_{S, LB}^{2}$, where *c*, $\mu$, and $\omega_{S, LB}$ are the speed of light, atomic mass per unit area, and frequencies of the shear and layer breathing modes, respectively.^[6,7]^ The masses of sulfur and rhenium atoms are $5.3\times{10}^{-26}$ kg and $3.1\times{10}^{-25}$ kg, respectively.^[8]^ The atomic mass per unit area, $\mu$, of monolayer ReS_2_ is calculated to be $4.5\times{10}^{-6}\mathrm{kg}m^{-2}$. Consequently, interlayer force constants can be expressed as $K_{S, LB}=8.0\times\omega_{S, LB}^{2}\times{10}^{16} N m^{-3}$. In our case, the $K_{S}$ and $K_{LB}$ values of the exfoliated bilayer ReS_2_ were estimated to be $17.0\times{10}^{18} N m^{-3}$ and $69.0\times{10}^{18} N m^{-3}$, respectively, which were in excellent agreement with the previously reported values.^[2,9]^


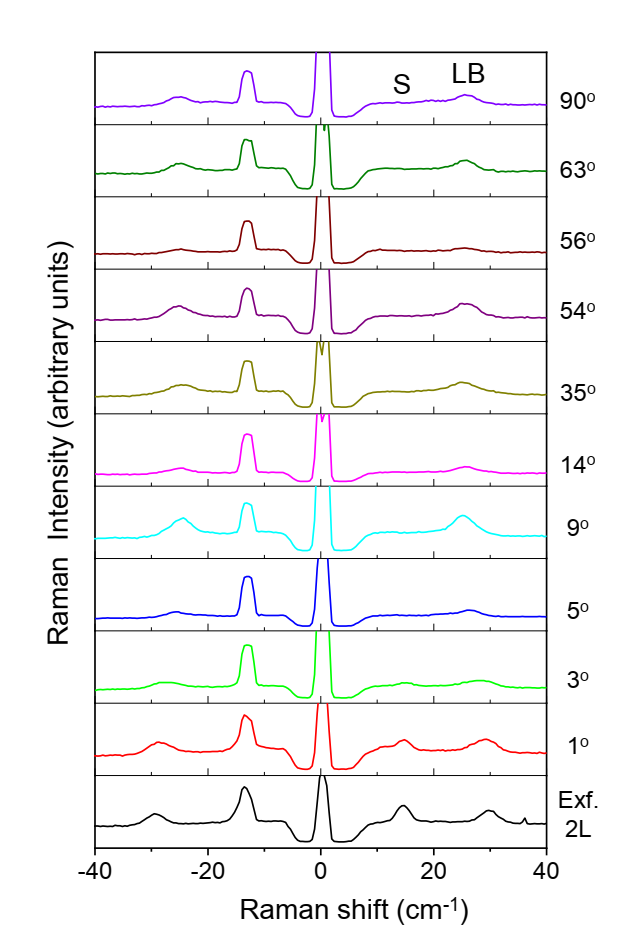


**Figure S5.** Low-frequency Raman spectra of tBL ReS_2_ at all twist angles studied. The S mode (LB mode) corresponds to the in-plane (out-of-plane) mode.


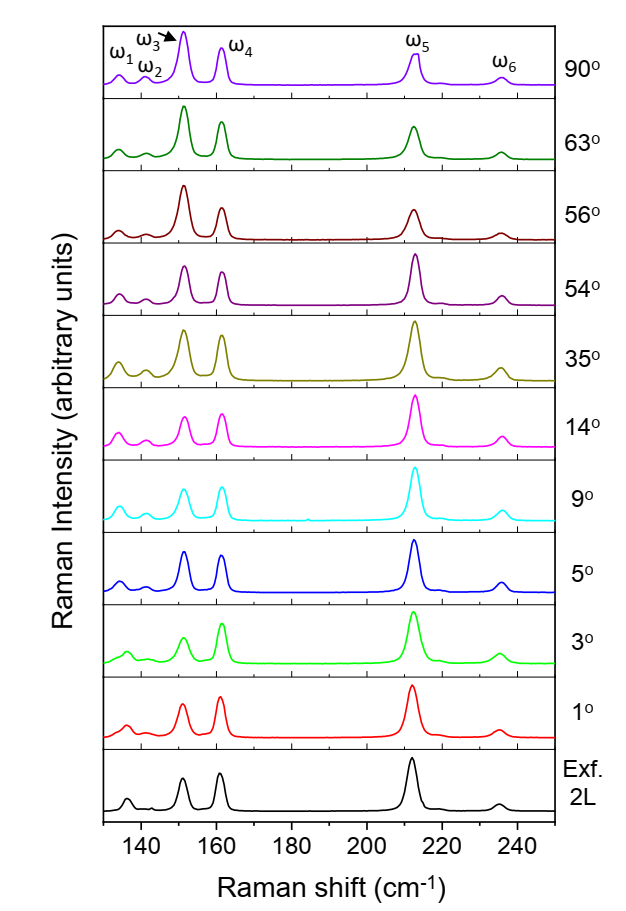


**Figure S6.** High-frequency Raman spectra of the tBL ReS_2_ for all twist angles studied.


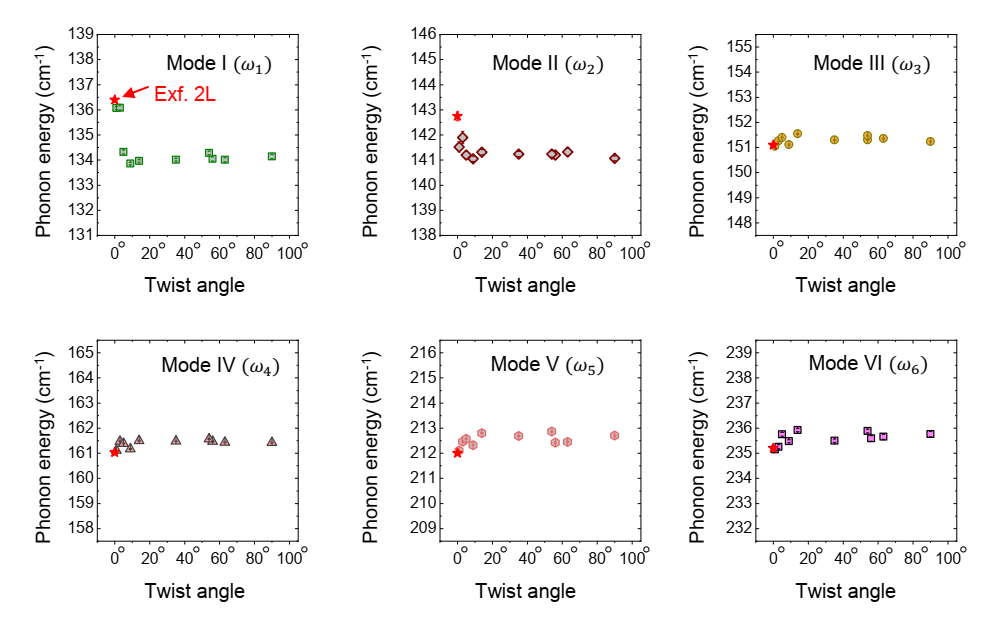


**Figure S7.** Plots of phonon energies of high-frequency Raman modes vs. twist angles.


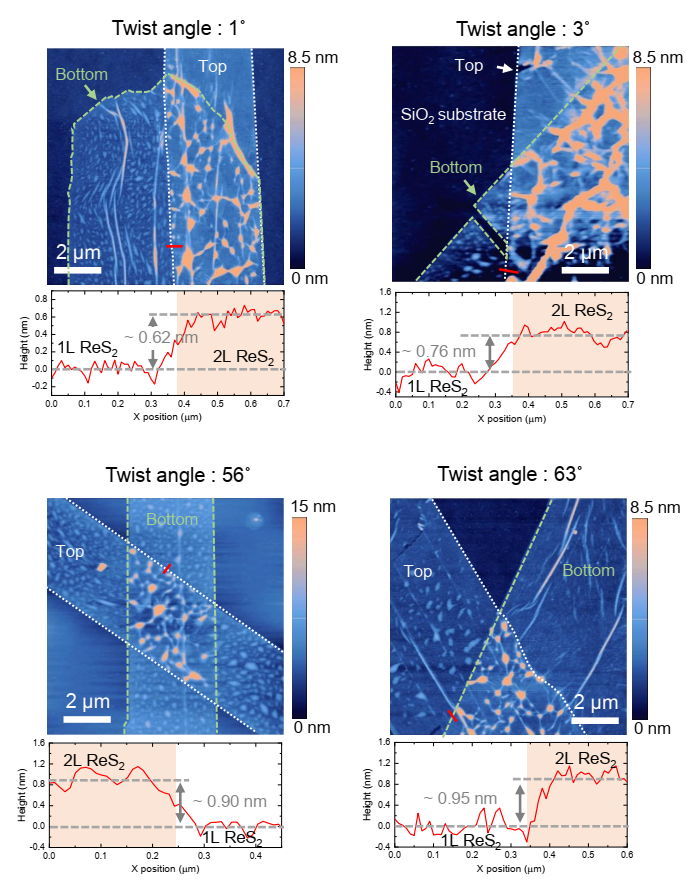


**Figure S8**. Atomic force microscope (AFM) measurement of four tBL ReS_2_ samples for the twist angle of 1°, 3°, 56°, and 63°. The heights of the top ReS_2_ monolayer from bottom ReS_2_ monolayer were estimated based on the AFM line profiles and the values are shown in the bottom panels, which were consistently higher for large angle twist angles (56° and 63°) samples than those of small twist angles (1° and 3°) samples. AFM results suggest that the interlayer distance between the top and bottom monolayers are smaller for small twist angles than for large twist angles, and considering that the stronger interlayer coupling means the less interlayer distance in bilayer TMDs ^[5,6]^ our AFM result is consistent with the results of our PL and Raman studies.

**Electrical transport measurement**

In order to investigate the effect of interlayer coupling in twisted bilayer ReS_2_ on its transport behavior, we fabricated a device, as shown in **Figure S9a-c**. After carefully adjusting the angle between the top and bottom layers of ReS_2_, the channel of twisted bilayer ReS_2_ was patterned using a reactive ion etcher and electron beam lithography. This was followed by metallization using Cr and Au. We fabricated three devices with bilayer ReS_2_ at different commensuration angles between the top and bottom layers, i.e., 0°, 1°, and 6°.

Prior to estimating carrier mobility, we characterized the transfer curves, which were further differentiated with respect to V_GS_ to extract the field-effect mobility $(\mu_{FE})$, as shown in Figure. S9d, S9e and S9f. The field-effect mobility of the intrinsic bilayer (0°) at 300 K was approximately $\mu_{FE} = 8 {cm}^{2}/V\cdot s$, which exceeds the value reported for a field-effect transistor (FET) with graphene electrodes.^[10]^ When the top and bottom layers were misaligned by 1°, the field-effect mobility showed a slight increase at 300 K. The field-effect mobility does not change much at the twisting angle of 1° at 100 K and 200 K.

Notably, Figure S9d, S9e and S9f demonstrates that the field-effect mobility decreases significantly when the twisting angle is increased to 6°, dropping to approximately $\mu_{FE} = 1 {cm}^{2}/V\cdot s$ at 300 K. A similar dependence of mobility on the twisting angle 6° was observed when the device was characterized at 100 K and 200 K. These transport study results are highly consistent with optical measurements, providing strong evidence of the demise or severe weakening of interlayer interaction at 6°.

The observed higher carrier mobility at small twist angles can be attributed to strong interlayer interaction, which allows the carriers to hop from the high-resistance layer to the lower-resistance layer. However, when the layers are not coupled, the carrier conduction remains only on the same layer.

**Figure S9.** (a,b,c) Optical image of bilayer ReS_2_ field-effect transistor twisted by 0°, 1°, and 6°. Field-effect mobility of bilayer ReS_2_ twisted by 0°, 1°, and 6° measured at (d) 100 K (e) 200 K and (f) 300 K. All scale bars in the optical image are 20 μm.


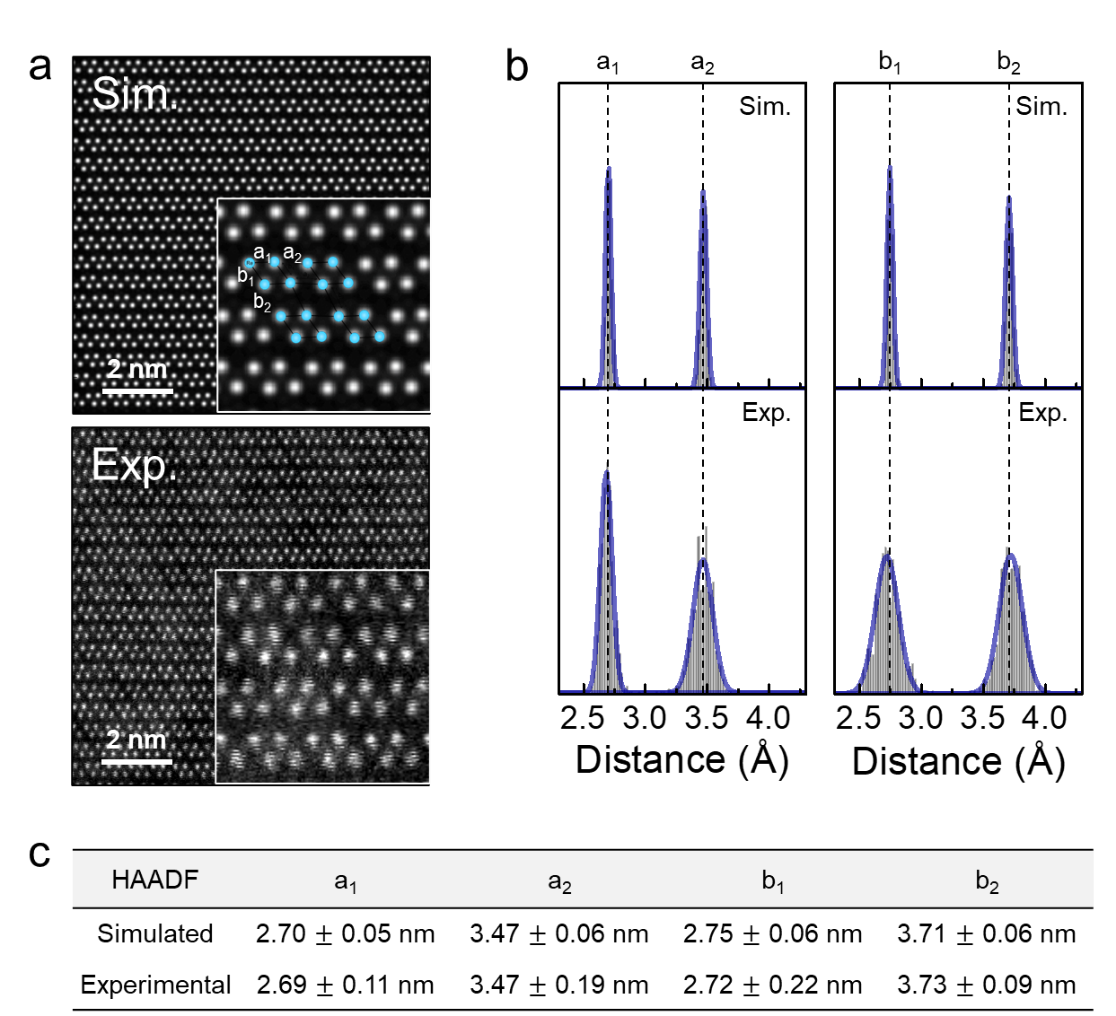


**Figure S10.** (a) Quantitative comparison of the simulated (top) and experimental (bottom) HAADF STEM images of 1T*'* ReS_2_ monolayer. Insets are magnified STEM images showing the one-to-one correspondence with the 1T*'* ReS_2_ atomic model. (b**)** Histograms showing the distributions of the measured (projected) bond lengths (a_1_, a_2_, b_1_, and b_2_) between Re atoms, which form an array of rhombic shapes. (c) List of the measured values of the four parameters from the histogram analysis. The bond lengths of Re atoms measured from the experimental HAADF STEM image correspond to those measured from the simulated image.

**Band structure and DOS of untwisted bilayer ReS_2_**

**
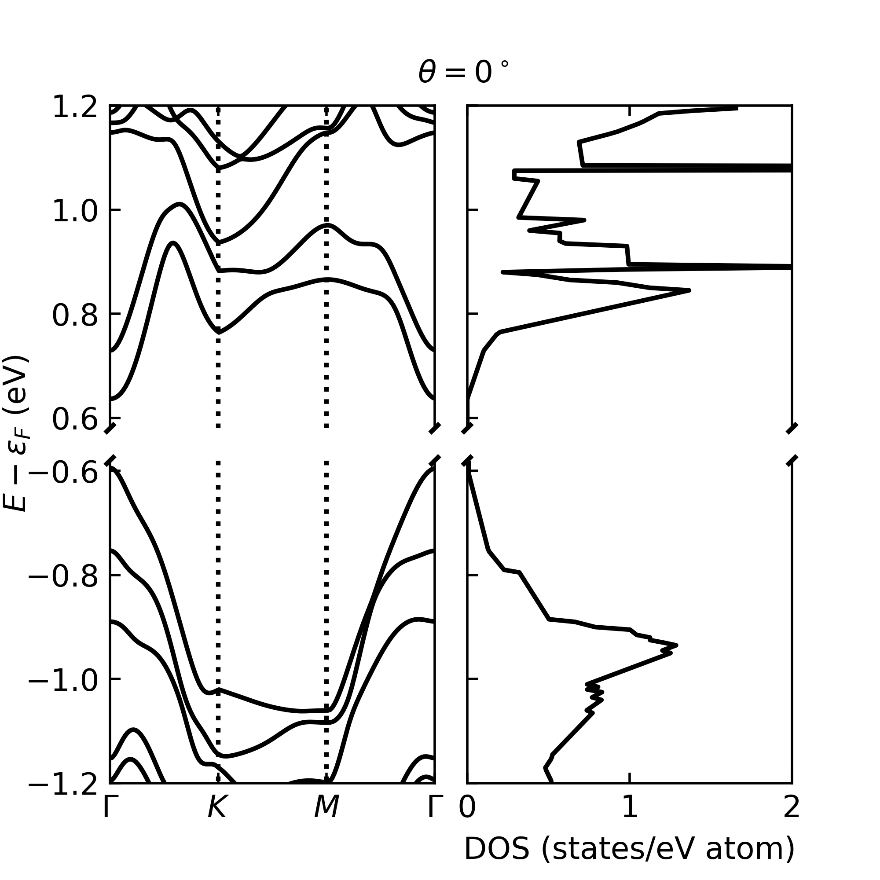
**

**Figure S11.** Band structure and DOS of untwisted bilayer ReS_2_, for comparison with its tBl counterparts at various angles. Small twist angles such as θ = 9.34° greatly decreases the bandwidth of the valence and conduction bands, and decreases the DOS associated with those bands by as much as 75%.

**References**

[1] D. A. Chenet, O. B. Aslan, P. Y. Huang, C. Fan, A. M. Van Der Zande, T. F. Heinz, J. C. Hone, *Nano Lett.* **2015**, *15*, 5667.

[2] R. He, J. A. Yan, Z. Yin, Z. Ye, G. Ye, J. Cheng, J. Li, C. H. Lui, *Nano Lett.* **2016**, *16*, 1404.

[3] Y. Cao, Y. Sun, H. Yang, L. Zhou, Q. Huang, J. Qi, P. Guan, K. Liu, R. Wang, *Nano Lett.* **2023**, *23*, 1211.

[4] Y. Choi, K. Kim, S. Y. Lim, J. Kim, J. M. Park, J. H. Kim, Z. Lee, H. Cheong, *Nanoscale Horizons* **2020**, *5*, 308.

[5] F. Kong, H. Wang, Y. Tong, L. Zhang, Y. Zhang, X. Han, K. Liu, J. Dai, H. Huang, C. Sun, L. Pan, D. Li, *ACS Nano* **2024**, *18*, 13899.

[6] S. Huang, L. Liang, X. Ling, A. A. Puretzky, D. B. Geohegan, B. G. Sumpter, J. Kong, V. Meunier, M. S. Dresselhaus, *Nano Lett.* **2016**, *16*, 1435.

[7] X. F. Qiao, J. Bin Wu, L. Zhou, J. Qiao, W. Shi, T. Chen, X. Zhang, J. Zhang, W. Ji, P. H. Tan, *Nanoscale* **2016**, *8*, 8324.

[8] T. Prohaska, J. Irrgeher, J. Benefield, J. K. Böhlke, L. A. Chesson, T. B. Coplen, T. Ding, P. J. H. Dunn, M. Gröning, N. E. Holden, H. A. J. Meijer, H. Moossen, A. Possolo, Y. Takahashi, J. Vogl, T. Walczyk, J. Wang, M. E. Wieser, S. Yoneda, X. K. Zhu, J. Meija, *Pure Appl. Chem.* **2022**, *94*, 573.

[9] E. Lorchat, G. Froehlicher, S. Berciaud, *ACS Nano* **2016**, *10*, 2752.

[10] M. Liu, L. Zhang, J. Liang, X. Li, Y. Dong, C. Zou, Y. Yang, K. Yang, S. Huang, *Phys. B Condens. Matter* **2019**, *554*, 35.
